# Comparison of transportation related injury mechanisms and outcome of young road users and adult road users, a retrospective analysis on 24,373 patients derived from the TraumaRegister DGU^®^

**DOI:** 10.1186/s13049-017-0401-1

**Published:** 2017-06-14

**Authors:** Thomas Brockamp, Uli Schmucker, Rolf Lefering, Manuel Mutschler, Arne Driessen, Christian Probst, Bertil Bouillon, Paola Koenen, Working Group Injury Prevention of the German Trauma Society (DGU), Committee on Emergency Medicine, Intensive Care and Trauma Management of the German Trauma Society (Section NIS), The TraumaRegister DGU

**Affiliations:** 1 Department of Trauma and Orthopedic Surgery, Cologne-Merheim Medical Center (CMMC), University of Witten/Herdecke, Ostmerheimer Str. 200, 51109 Cologne, Germany; 2 Working Group of Injury Prevention of the German Trauma Society, The German Trauma Society, Straße des 17. Juni 106-108, 10623 Berlin, Germany; 3 Depatment for Trauma Surgery, University Hospital Regensburg, Franz-Josef-Strauss-Allee 11, 93042 Regensburg, Germany; 4 AUC - Academy for Trauma Surgery, Wilhelm-Hale-Str. 46b, 80639 Munich, Germany; 5 Institute for Research in Operative Medicine (IFOM), University of Witten/Herdecke, Cologne-Merheim Medical Center (CMMC), Ostmerheimer Str. 200, 51109 Cologne, Germany; 6 Committee on Emergency Medicine, Intensive Care and Trauma Management of the German Trauma Society (Section NIS), The German Trauma Society, Straße des 17. Juni 106-108, 10623 Berlin, Germany; 7 University of Witten/Herdecke, Faculty of Health - School of Medicine, Cologne-Merheim Medical Center (CMMC), Ostmerheimer Str. 200, 51109 Cologne, Germany

**Keywords:** Trauma, Youth, Adult, Mechanism of injury, Injury pattern

## Abstract

**Background:**

Most young people killed in road crashes are known as vulnerable road users. A combination of physical and developmental immaturity as well as inexperience increases the risk of road traffic accidents with a high injury severity rate. Understanding injury mechanism and pattern in a group of young road users may reduce morbidity and mortality. This study analyzes injury patterns and outcomes of young road users compared to adult road users. The comparison takes into account different transportation related injury mechanisms.

**Methods:**

A retrospective analysis using data collected between 2002 and 2012 from the TraumaRegister DGU® was performed. Only patients with a transportation related injury mechanism (motor vehicle collision (MVC), motorbike, cyclist, and pedestrian) and an ISS ≥ 9 were included in our analysis. Four different groups of young road users were compared to adult trauma data depending on the transportation related injury mechanism.

**Results:**

Twenty four thousand three hundred seventy three, datasets were retrieved to compare all subgroups. The mean ISS was 23.3 ± 13.1. The overall mortality rate was 8.61%. In the MVC, the motorbike and the cyclist group, we found young road users having more complex injury patterns with a higher AIS pelvis, AIS head, AIS abdomen and AIS of the extremities and also a lower GCS. Whereas in these three sub-groups the adult trauma group only had a higher AIS thorax. Only in the group of the adult pedestrians we found a higher AIS pelvis, AIS abdomen, AIS thorax, a higher AIS of the extremities and a lower GCS.

**Discussion:**

This study reports on the most common injuries and injury patterns in young trauma patients in comparison to an adult trauma sample. Our analysis show that in contrast to more experienced road users our young collective refers to be a vulnerable trauma group with an increased risk of a high injury severity and high mortality rate. We indicate a striking difference in terms of the region of injury and the mechanism of injury when comparing the young versus the adult trauma collectives.

**Conclusions:**

Young drivers of cars, motorbikes and bikes were shown to be on high risk to sustain a specific severe injury pattern and a high mortality rate compared to adult road users. Our data emphasize a characteristic injury pattern of young trauma patients and may be used to improve trauma care and to guide prevention strategies to decrease injury severity and mortality due to road traffic injuries.

## Background

Death and disability due to road traffic accidents especially affect young road users with a disproportionate number occurring in developing countries. Although the child injury death rate is much lower among children from developed countries, injuries are still a major cause of death, accounting for about 40% of all child deaths [1–3]. Road traffic injuries alone are the leading cause of death among 15 to 19-year-olds and the second leading cause among 10 to 14-year-olds [1, 2]. It is known that transportation related injury mechanisms (Motor vehicle collisions (MVC), motorbike, cyclists and pedestrians) do frequently result in a high Injury Severity Score (ISS) especially in the group of young road users [1, 4–8]. A combination of physical and developmental immaturity among children, and inexperience and youth-related lifestyles further increase the risk to road traffic collisions. Understanding the injury pattern of road users in different age groups is critical to improve trauma care and to guide prevention strategies to reduce high injury severity and mortality rates [9]. Today the measure of road safety is not only the number of people that are killed by accidents but moreover the severity of injury.

With regard to the disability adjusted life years lost trauma remains a significant socio-economic problem. Thus, decreasing the burden of injury remains a challenge for public health in the next century [1]. The WHO (World Health Organization) and other global organizations have taken a strong stand against this public health threat, with resources, research, guidance documents, global resolutions and injury prevention strategies to curb the road traffic injury problem [10]. Promotion strategies including economic and retailer interventions, alcohol taxation, reducing alcohol availability, legal and legislative strategies, and strategies addressing the servers of alcohol have been shown to reduce driving under the influence of alcohol [11, 12]. Prevention strategies can reduce the number of incidents or limit its injurious consequences.

Although there have been recent national assessments of pediatric trauma using hospital discharge information or trauma databases, these studies have analyzed patterns of injures in different age groups [5, 13]. To our knowledge, not much is known about the injury pattern of certain injury mechanisms in different groups of young road users (3–22 years) compared to a large group of adult road users. The aim of this study was to find out about differences regarding the mechanism of injury related to injury severity and mortality in young and adult trauma patients including trauma data derived from the TraumaRegister DGU® (TR-DGU).

## Methods

### TraumaRegister DGU®

The TraumaRegister DGU® of the German Trauma Society (Deutsche Gesellschaft für Unfallchirurgie, DGU) was founded in 1993. The aim of this multi-center database is a pseudonymised and standardized documentation of severely injured patients. Data are collected prospectively in four consecutive time phases from the site of the accident until discharge from hospital: A) Pre-hospital phase, B) Emergency room and initial surgery, C) Intensive care unit and D) Discharge. The documentation includes detailed information on demographics, injury pattern, comorbidities, pre- and in-hospital management, course on intensive care unit, relevant laboratory findings including data on transfusion and outcome of each individual. The inclusion criterion is admission to hospital via emergency room with subsequent ICU/ICM care or reach the hospital with vital signs and die before admission to ICU.

The infrastructure for documentation, data management, and data analysis is provided by AUC - Academy for Trauma Surgery (AUC - Akademie der Unfallchirurgie GmbH), a company affiliated to the German Trauma Society. The scientific leadership is provided by the Committee on Emergency Medicine, Intensive Care and Trauma Management (Sektion NIS) of the German Trauma Society. The participating hospitals submit their pseudonymised data into a central database via a web-based application. Scientific data analysis is approved according to a peer review procedure established by Sektion NIS.

The participating hospitals are primarily located in Germany (90%), but a rising number of hospitals of other countries contribute data as well (at the moment from Austria, Belgium, China, Finland, Luxembourg, Slovenia, Switzerland, The Netherlands, and the United Arab Emirates). Currently, approx. 25,000 cases from more than 600 hospitals are entered into the database per year. Participation in TraumaRegister DGU® is voluntary. For hospitals associated with TraumaNetzwerk DGU® however, the entry of at least a basic data set is obligatory for reasons of quality assurance.

The present study is in line with the publication guidelines of the TraumaRegister DGU® and registered as TR-DGU project ID 2013-010.

### Study sample

In the present work datasets of 24,373 patients from the TR-DGU that were collected between 2002 and 2012 were retrospectively analyzed and used for data analysis. Only patients with a transportation related injury (MVC, motorbike, cyclist, and pedestrian) and an ISS ≥ 9 were included and are henceforth referred to as study sample (excluding trauma not related to road use). The transportation related injury mechanism are those defined by prior studies and are known as the leading causes for injuries in the western world [14, 15].

**Fig. 1:**
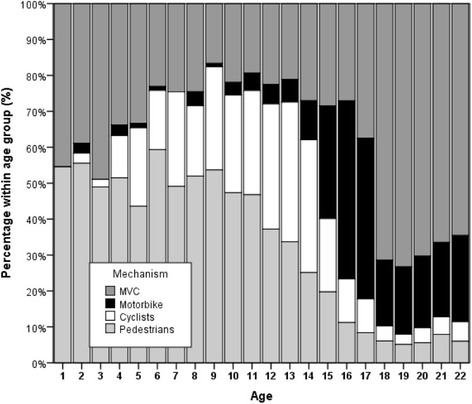
Absolut number of cases per age group (Injuries of cars; motorbikes; cyclists and pedestrians)

**Fig. 2:**
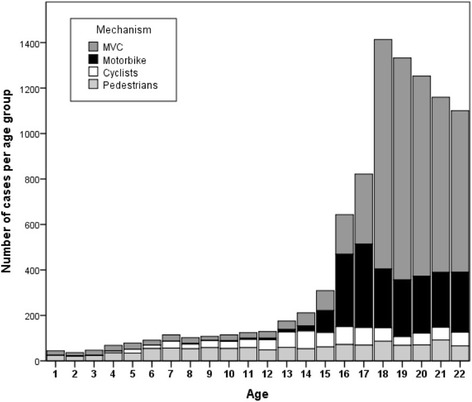
Relative number of cases per age group (Injuries of cars; motorbikes; cyclists and pedestrians)

The study sample was first divided in two groups: A young trauma group aged 3–22 years (*n* = 7641) and an adult group aged 25–50 years (*n* = 16,732). In the young trauma group, we then defined four subgroups, one for each injury mechanism. In our analysis, MVC were the leading mechanism of injury for young road users between 18 and 22 years, Motorbike accidents were the leading mechanism of injury for 15–22 years. As we also wanted to include the youngest road users we looked at cyclists and pedestrians between 4 and 17 and 3–15 years of age respectively. Each subgroup was compared to an adult group aged 25–50 years (Figs. 1 and 2).

### Statistical analysis

The statistical analysis of this study was based upon the database of the TR-DGU (2002 to 2012). Data are presented as mean with standard deviation (±SD) for continuous variables and as percentages for incidence rates. Statistical analysis was performed applying standard statistical software (SPSS Version 17.0, SPSS, Chicago, IL, USA).

## Results

The study sample consisted of 24,373 cases with a mean age of 31.6 ± 11.5 years and a male predominance (76% of study sample). In the young MVC sample the mean age was 20 ± 1 years compared to 37 ± 8 years in the adult MVC sample (motorbike sample 19 ± 2 years/38 ± 8 years; bicycle sample 13 ± 1/40 ± 8; pedestrian sample 10 ± 4/39 ± 8). The mean ISS was 23.3 ± 13.1. The overall mortality rate was 8.61% (*n* = 1982). The mortality rate was significantly higher in the group of the young road users that were involved in MVC, motorbike accidents or in accidents as a cyclist. (Mortality rate MVC: young sample 9.4% versus 7.8% in the adult trauma sample (*p* = 0.003); Mortality rate motorbike: 8.4% versus 6.8% (*p* = 0.03); Mortality rate cyclist group: 8.7% versus 6.2% (*p* = 0.038)). Only adult pedestrians had a higher mortality rate compared to the young pedestrian group (13.0% versus 6.8%, *p* < 0.001).

Table 1 provides a demographic overview of the study sample plus injury pattern and physiological baseline data. In the MVC, the motorbike and the cyclist group, we found the young road users to have a more complex injury pattern with a higher AIS pelvis, AIS head, AIS abdomen and AIS of the extremities and a lower GCS. Whereas in these three groups the adult trauma group only had a higher AIS thorax. Vice versa only in the group of the adult pedestrians did we find a higher AIS pelvis, AIS abdomen, AIS thorax, a higher AIS extremities and a lower GCS. In the group of the young pedestrians we only found a higher AIS head.

**Table 1:** Demographic data

Variable		MVC		Motorbike		Cyclist		Pedestrian	
		Youth (18–22y)	Adult (25–50y)	Youth (15–22y)	Adult (25–50y)	Youth (4–17y)	Adult (25–50y)	Youth (3–15y)	Adult (25–50y)
	n	4350	7789	2048	5622	597	1857	646	1464
Age (years)	Mean ± SD	20 ± 1	37 ± 8	19 ± 2	38 ± 8	13 ± 3	40 ± 8	10 ± 4	39 ± 8
Male	n (%)	2995 (69.3)	5286 (68.2)	1816 (89.1)	5003 (89.4)	436 (73.0)	1351 (73.0)	399 (61.8)	1039 (71.3)
SBP (≤90 mmHg)	n (%)	633 (17.9)	1088 (17.2)	276 (16.4)	730 (15.8)	78 (17.4)	167 (11.5)	104 (20.1)	237 (19.7)
GCS (≤8)	n (%)	1122 (30.3)	1516 (22.7)	410 (23.2)	902 (18.5)	141 (29.0)	331 (21.6)	174 (30.4)	431 (33.8)
AIS (Head ≥ 3)	n (%)	1918 (44.1)	2703 (34.7)	670 (32.7)	1450 (25.8)	337 (56.4)	1037 (55.8)	332 (51.4)	717 (49.0)
AIS (Thorax ≥ 3)	n (%)	2247 (51.7)	4462 (75.3)	887 (43.3)	3160 (56.2)	166 (27.8)	680 (36.6)	182 (28.2)	627 (42.8)
AIS (Abdomen ≥ 3)	n (%)	849 (19.5)	1442 (18.5)	398 (19.4)	945 (16.8)	100 (16.8)	159 (8.6)	70 (10.8)	212 (14.5)
AIS (Pelvis ≥ 3)	n (%)	1020 (23.4)	1671 (21.5)	341 (16.7)	1133 (20.2)	65 (10.9)	176 (9.5)	137 (21.2)	396 (27.0)
AIS (Extremities ≥ 3)	n (%)	1850 (42.5)	3187 (40.9)	1046 (51.1)	2666 (47.4)	148 (24.8)	304 (16.4)	252 (39.0)	650 (44.4)
ISS	Median (*)	22 (25 ± 14)	21 (24 ± 13)	18 (23 ± 14)	20 (23 ± 13)	17 (21 ± 12)	18 (21 ± 11)	17 (21 ± 13)	22 (26 ± 14)
NISS	Median (*)	26 (29 ± 15)	24 (28 ± 15)	22 (28 ± 15)	24 (28 ± 14)	22 (25 ± 15)	22 (27 ± 14)	22 (26 ± 15)	27 (30 ± 17)
LOS on ICU (days)	Median (*)	4 (8 ± 10)	4 (8 ± 11)	3 (7 ± 10)	4 (8 ± 12)	3 (6 ± 10)	3 (7 ± 10)	2 (5 ± 8)	4 (9 ± 13)
LOS in hospital (days)	Median (*)	15 (20 ± 21)	17 (23 ± 24)	16 (21 ± 21)	19 (26 ± 28)	10 (15 ± 16)	12 (17 ± 20)	11 (14 ± 14)	19 (24 ± 25)
Mortality	n (%)	409 (9.4)	609 (7.8)	172 (8.4)	390 (6.8)	52 (8.7)	116 (6.2)	44 (6.8)	190 (13.0)

Data are presented as mean or as percentage. ISS, NISS, LOS ICU and LOS hospital are presented as median. *SBP* Systolic Blood Pressure, *GCS* Glasgow Coma Scale, *AIS* Abbreviated Injury Scale, *ISS* Injury Severity Score, *NISS* New Injury Severity Score, *ICU* Intensive Care Unit, *LOS* Length of stay, *MVC* Motor vehicle collision. (*) Additionally, the ISS, NISS, LOS ICU and LOS in hospital are presented as mean ± SD

**Fig. 3:**
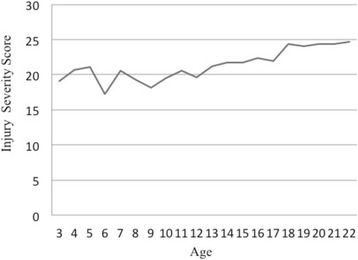
ISS depending on age

Figures 1 and 2 illustrate the absolute and relative numbers of cases in which young car drivers, motorbike drivers, cyclists and pedestrians were involved regarding age. Furthermore, Fig. 3 shows the ISS depending on age.

## Discussion

This study reports on the most common injuries and injury patterns in young trauma patients in comparison to an adult trauma sample. Our analysis show that in contrast to more experienced road users our young collective refers to be a vulnerable trauma group with an increased risk of a high injury severity and high mortality rate. We indicate a striking difference in terms of the region of injury and the mechanism of injury when comparing the young versus the adult trauma collectives.

We found that road users under the age of 21 years who were involved in an accident while driving in a car, with a motorbike or on a bike had a significantly higher AIS head, AIS abdomen, AIS extremities as well as a lower GCS. The adult trauma collective had more severe thorax injuries. On the other hand, adult pedestrians were shown to have more complex injuries with a higher AIS head, thorax, abdomen, extremities and a lower GCS and a significantly higher mortality rate compared to the young pedestrian group.

However, in 2007 Buschmann et al. published a retrospective analysis and compared 517 pediatric trauma patients against 11,025 adult trauma patients. They found the young trauma collective to have more severe injuries to the head and that the amount of severe injuries to the thorax increases with age [16]. This can also be confirmed by our data.

Bayreuther et al. found a decline in the proportion of children with head injuries and an increase in the proportion with limb injuries with increasing age [17]. Reichmann et al. also showed similar results [17, 18]. They analyzed data of pediatric and adult trauma patients and found more severe injuries to the thorax and less severe injuries to the head the older the patient was.

Based on our data we can draw the conclusion, that a young and inexperienced car driver/occupant, motorbike driver or cyclist has a significantly higher risk to sustain more severe injuries to the head, abdomen and extremities which results in a higher mortality rate compared to adult road users. The more complex and more severe kind of injury in the young trauma group could be due to different injury mechanisms compared to those of more experienced drivers. Factors like over estimation, risk-awareness and foresighted driving influence the impact of a crash and the impact to the human body during the crash. Novice drivers do not have the ability to evaluate and assess difficult and dangerous situations that might appear on the roads.

Because trauma is the leading cause of death among young road users; we think that it is important to understand major mechanisms of trauma especially in this trauma population [1, 2, 4, 6, 7]. Analyzing injury mechanism and pattern is crucial to better direct prevention efforts and interventions. Reducing the number of road traffic injuries and deaths as well as the severity of injury patterns is an important issue. A number of factors increase the likelihood of road traffic injuries occurring, not only among young people, but also in the general population. These include speed, lack of helmet use, lack of seat-belt and child restraint use, drinking and driving, and lack of conspicuity [9].

Today a lot of injury prevention strategies and methods have been set up to enlighten young road users about the consequences of trauma. Especially educational measures, community based measures as well as legislative measures were set up to reduce accidental injuries in the young population. Evidence of the effectiveness for each program is rare. The most effective measures seem to be legislative. While the results reported from community based approaches are encouraging, there is little evidence that purely educational measures reduce injury rates in the short term [19, 20].

The P.A.R.T.Y. program is an example for an educational measure with a focus on novice drivers. The program is held in a trauma hospital and the participants face real trauma patients and their history of injury. There is evidence that this program can have a positive effect on young road users [21, 22]. Due to a limit of sponsoring, financing and staff, it is important to implement appropriate strategies with a high grade of impact.

In addition to the need of prevention measures, trauma centers have been shown to improve outcome of severely injured patients [23, 24]. Over the past decade, there has been much debate regarding to pediatric trauma outcomes and their association with different types of trauma centers. Several recent studies have concluded that injured children treated at pediatric trauma centers have better outcomes and are more likely to survive compared with those treated at adult trauma centers [25].

Limitations of the present study are the same as those in retrospective studies utilizing registry data. The data is furthermore limited, as the TR-DGU has been designed to register severely and/or multiply injured patients requiring ICU admission only. Because of this, no data are available about patients with minor injuries. One important step in analyzing our data was the definition of different age groups in the young and in the adult population. Due to this we analyzed all data of the TR-DGU of each injury mechanism (MVC, Motorbike, Bike, Pedestrian) and looked at all ages in which the respective mechanism of injury is a leading mechanism of injury. We did not want to simply take one group of pediatrics (e.g. 16–25 years) and compare it with an adult group, because we also wanted to look at the youngest road traffic participants and therefore used the pedestrian and cyclist group. Using different age groups for data analysis might give different results. However, as we looked at different constellations regarding all age groups, we can say that our main results would not have been notably influenced. Moreover, our data do not give information about the kind of injury. In example, if a car driver crashed into another car or against a tree. And we can also not differentiate between car driver and car occupant. This might also be a limitation in our dataset.

## Conclusions

Several conclusions can be drawn from these data. Young drivers and occupants of cars, motorbikes and bikes are at high risk to sustain multiple injuries with a high injury severity and mortality rate. Improvements should be done by legislation but also by prevention measures and in the supply of young trauma patients. However, data about injury pattern and the kind of injury can provide information for quality assessment, for staff and for targeting injury prevention programs.

## Abbreviations

AIS: Abbreviated injury scale
GCS: Glasgow coma scale
ICU: Intensive care unit
ISS: Injury severity score
MVC: Motor vehicle collision
TR-DGU: TraumaRegister DGU®
WHO: World Health Organization
